# Child Maltreatment and the Child Welfare System as Environmental Factors in the International Classification of Functioning

**DOI:** 10.3389/fresc.2021.710629

**Published:** 2022-01-17

**Authors:** Katherine Kim, Corinne Moss, Jane Jungyoon Park, Christine Wekerle

**Affiliations:** ^1^Department of Pediatrics, McMaster University, Hamilton, ON, Canada; ^2^Temerty Faculty of Medicine, University of Toronto, Toronto, ON, Canada

**Keywords:** child welfare, foster care, child maltreatment, mental health, functioning, youth, adolescence, disability

## Abstract

The WHO defines child maltreatment as any form of neglect, exploitation, and physical, emotional, or sexual abuse, committed against children under the age of 18. Youth involved in the child welfare system report more maltreatment experiences and environmental turbulence (e.g., number of moves, caseworkers), placing them at greater risk for poorer physical and mental health. The International Classification of Functioning, Disability, and Health (ICF) provides a framework to describe health conditions and severity of disabilities for an individual and/or group in the context of environmental factors. The Maltreatment and Adolescent Pathways (MAP) study is a longitudinal study, assessing self-reports on variables (e.g., child maltreatment history, trauma symptoms, dating violence, and substance use) of youth in an urban child protection service system. This study focuses on 11 of the 24 MAP publications that pertain to health and functioning, which can be considered applicable to the ICF framework, following established linking rules. The purpose of this study is to analyze these MAP sub-studies, with maltreatment and involvement in the child welfare system as environmental factors that impact the functioning of child welfare-involved youth. Findings indicate significant relationships across environmental factors (i.e., child maltreatment histories, child welfare system involvement), health conditions (i.e., trauma symptomatology, psychological distress, intellectual disabilities), and functioning problems (i.e., substance use, adolescent dating violence, sexual risk-taking, coping motives, sleep problems). The interrelated nature of these factors in the MAP sub-studies suggests the value of the ICF model to a holistic health view of use to practitioners supporting system-involved youth, clarifying unattended environmental factors in guiding service provision for foster care and/or maltreated youth.

## Introduction

Child maltreatment is a significant public health issue. While most countries have child welfare systems, many maltreated youths are undetected by practitioners, and practitioners experience hesitation in fulfilling mandatory reporting duties [e.g., (1)]. According to the WHO, child maltreatment is defined as: “All forms of physical and/or emotional ill-treatment, sexual abuse, neglect or negligent treatment or commercial or other exploitation, resulting in actual or potential harm to the child's health, survival, development, or dignity in the context of a relationship of responsibility, trust or power” [(2); refer to Table 1 for definitions of types]. In North America, the median prevalence of physical abuse was 24.3% for boys and 21.7% for girls. The prevalence of sexual abuse was 14.1% for boys and 20.4% for girls. Emotional abuse was 28.4% for boys and 23.8% for girls. Median rates of neglect differed substantially between girls (40.5%) and boys (16.6%) (4). It is well-recognised that poly-victimisation describes many youths in the child welfare system and is related to poorer outcomes (5). Studies have demonstrated a dose-response relationship, where an increased number of childhood maltreatment experiences correlated with elevated dysfunction (6, 7). While estimates of the cost to the victims and their communities are unquantifiable, the service system costs have been estimated. Based on official reporting data, the cost of non-fatal child maltreatment in 2015 was estimated to be $830,928 per case, and fatal outcomes cost $16.6 million in the United States (8). Furthermore, the total economic burden in the United States for substantiated incident cases was $428 billion in 2015 (8). It is clearly established that maltreatment yields lifespan impact to physical, mental, and financial health.

**Table 1:** Definitions of childhood maltreatment types (3).

**Term**	**Definition**
Physical abuse	“A caregiver inflicting physical harm or engaging in actions that create a high risk of harm”
Emotional abuse	“Inflicting emotional harm through the use of words or actions”
Sexual abuse	“Any action with a child that is done for the sexual gratification of an adult or significantly older child”
Neglect	“Failure to meet a child's basic physical, emotional, educational, and medical needs”

The victim of child maltreatment is probabilistically more likely to face challenges, with greater risk of poverty, and the likelihood of finding themselves homeless, couch-surfing within their communities, or street-involved (9). Living in such circumstances brings another level of harm risk, such as increased access to a greater array of substances, opportunities to make money by selling substances, increased likelihood of violence victimisation, as well as vulnerability to recruitment for sex trafficking (10). Child maltreatment is associated with multiple negative outcomes, most notably in the areas of mood disorders (e.g., anxiety, depression), substance use problems, relationship violence, and chronic physical health conditions or non-communicable diseases, as stress pathways are triggered with cascading potentials (11–16). In addition to adverse events being more prominent, there is also a lack of positive or benevolent childhood events (e.g., presence of a positive person in which to confide, opportunities for school achievement, secure parent-child attachment relationships) (17). There has been much discussion over poverty risk and child neglect, as families with socioeconomic disadvantage come to the attention of the child welfare system to a greater extent than more affluent families (18). If child welfare system engagement creates a safety net around the child, mandatory reporting may be considered part of prevention planning for re-victimisation risk (1). In over 30 countries, mandatory reporting laws direct suspected abuse and neglect events or risks thereof to the responsible child welfare authorities (19). The most comprehensive laws and policies are in the United States, Canada, and Australia, yet many other high-income countries do not have such laws as policy (e.g., Hong Kong, Germany, Hungary, etc.). Entry into the child welfare system may bring a wide range of financial, medical, and other social services to the child and/or family. As a foster care youth living in an out-of-home arrangement, there may be a linkage to a caring home and a stable caseworker, or there may be experiences of revictimization in care and a prolonged lack of permanency (20), multiple residential transitions (21, 22), as well as multiple changes in caseworkers (23). According to Waid et al. 40.8% of youth in foster care had experienced at least one placement change throughout 18 months (24). Furthermore, caseworker turnover rates have been reported to be 30–40% annually (25). However, the system-specific risk may be heightened when a youth “ages out of care” in adolescence or early adulthood, potentially without transition services and a social safety net.

### Maltreatment, Adolescence, and Child Welfare

According to the United States Department of Health and Human Services (USDHHS) Child Maltreatment 2019 report (26), infants (up to age 2) are the largest group for entry into foster care, representing 28% of all cases, or a rate of 26.7 per 1,000 children (27). Youths aged 12 and older are the next highest timeframe for coming into care (28). As of 2020, the number of youths in care in the United States is ~424,000 (29). The WHO defines adolescence from 10 to 19 years of age, which is noted as a critical development period. With the onset of puberty, increases in sex hormones contribute to rapid changes in physical growth, the brain, and behaviour (30). In terms of maltreatment types, adolescence is a higher time of sexual violation among females: ages 14 and above represented about 72% of sex trafficking cases in child welfare (27). Thus, it is important to focus on child sexual abuse (CSA) when considering the functioning of adolescents, in terms of risk, as well as in terms of its potential impact on the adolescent developmental task of becoming attuned to and involved in romantic relationships. Furthermore, adolescence is a known high period of cognitive development, wherein risk-taking, and abstract and strategic thinking becomes refined (31). Studies have highlighted the potential negative impact of stressful life experiences during adolescence, which may disrupt normal neural development and contribute to adolescent vulnerability to mental diseases, such as anxiety and/or depression (32–34). Among children investigated by child protection services (CPS) in the National Survey of Child and Adolescent Well-being (NSCAW) II, approximately half were noted as having developmental issues (35). As compared to children without disabilities, child welfare-involved children with intellectual disabilities were at greater risk of experiencing placement instability, challenges with adoption, and not being able to reunite with kin (36). Child welfare-involved youth are also at higher risk for re-victimisation in their dating relationships (37). Dating violence victimisation is associated with self-reported post-traumatic stress symptomatology (PTSS), drug use, and previous dating violence perpetration (37–39).

While entry into child welfare indicates a sentinel maltreatment risk event, there is a wide range of experiences that may occasion re-victimisation, such as visitation with kin or parents, experiences in the alternate care environment, street involvement, and the use of restraints in institutional care. This potential for further trauma needs to be regarded as an active “rule out” when child welfare youth are cared for in the medical home (40). As youth-reported maltreatment experiences have been shown to be higher than official recordings of victimisation, it is critical to ask youth directly about their victimisation experiences [e.g., (41)].

### The International Classification of Functioning, Disability, and Health

The International Classification of Functioning, Disability, and Health (ICF) by the WHO provides a standardised language and framework for describing an individual's health, functioning, and disability (42). The guiding document for definitions, categories, and codes for the ICF is the ICF Red Book (42). The ICF is a hierarchal classification, based on the biopsychosocial model which views functioning as an outcome of the dynamic interactions of both health conditions (e.g., diseases, disorders, and injuries) and contextual factors (e.g., social, child welfare system involvement) (42). The ICF is structured into two parts: (1) functioning and disability, and (2) contextual factors. Within these two parts are six levels ordered as a hierarchy, from general to more detailed and specific entities: components, domains, constructs, positive aspects, and negative aspects. Functioning and disability include these components: body functions (e.g., sleep, pain, and emotional functions) and structures (e.g., nervous, reproductive, and endocrine system), as well as activities (e.g., basic learning, self-care, and mobility) and participation (e.g., communication, interpersonal relationships, and education). Contextual factors include the components: personal (e.g., sex, age, and ethnicity) and environmental (e.g., technology, social support, and policies) factors (43). Domains, constructs, positive aspects, and negative aspects progressively detail the aspects of functioning. This study will focus on the components of functioning and disability and contextual factors.

As applied to an individual, the ICF can provide a snapshot in time that allows one to consider a wide array of variables to obtain a more holistic analysis of the individual's historical factors and current functioning. This model may be particularly beneficial in the context of child maltreatment. For example, youth with higher exposure to maltreatment report higher levels of chronic pain (44). Pain in identified locales, or chronic generalised pain, would appear in the ICF body functions domain. When encountering youth in chronic pain without a clear medical condition, the differential diagnosis may be to rule out child maltreatment history, noting most victims do not disclose (45). The ICF model would be more likely to detect this child maltreatment history as a barrier to functioning, due to its more holistic approach. The ICF is, therefore, well-suited to frame the “whole” youth in terms of inter-connecting domains of functioning, for example, multiple problems that may ensue from the presence of post-traumatic stress symptoms (PTSS) of arousal (e.g., sleep disturbance, sexual risk-taking/multiple partners), negative cognitions and mood (e.g., depression), avoidance (e.g., substance abuse) and re-experiencing (e.g., re-victimisation) (46). The principle of multi-finality (47) reflects that many outcomes may arise from a common event (e.g., maltreatment experiences as the abuse of power and negligence).

The application of the ICF to conceptualising or matching service needs within child welfare has been under-utilised. Most of the research in child welfare has focused on rehabilitation services, related to physical or cognitive disability, to the relative exclusion of mental health and related problems as disability or risk thereof. Only one child welfare study was found, to our knowledge, that explored disability (i.e., physical, medical, intellectual), as well as mental health conditions *via* the ICF framework (48). Indeed, the overlap with a physical and cognitive disability, and other areas of functioning compromise, is an area of research gap. It is critical to take into account disability in terms of complex trauma or ongoing mental health challenges, which facilitate other problems such as alcohol use and dating violence. As such, a focus on adolescent functioning within child welfare represents a valid start to considering the application and utility of an ICF approach, for integration across disability types, mental health problems, and environmental factors. It must also be considered that many child welfare system-involved youth show remarkable resilience (49). The progression through child welfare may involve case openings and closings before an out-of-home care option is sought permanently. Thus, the ICF application may be best understood in terms of distal or historical domains, compared to current or proximal influences.

In this study, we review a selection of studies that use data from the first comprehensive research study on child welfare system-involved youth in Canada, known as the Maltreatment and Adolescent Pathways (MAP) study. This longitudinal study captures data from adolescents in a large urban CPS system in Ontario, Canada, with study entry between ages 14 and 17, and followed up to 3 years. Most youth will exit the child welfare system in the age ranges of 16–18 years, with some extending support to age 21. By investigating these MAP sub-studies in the context of the ICF framework, we aim to explore the complex interactions of factors that affect the functioning and health of maltreated, system-detected youth and propose a model of distal and proximal considerations. Given the mobility of child welfare-involved youth and the rapidity with which most cases are closed or transferred to other services, a functioning-oriented approach seems essential to guide youths' health and well-being, wherever they are at in their trajectory through child welfare services. The goal herein was to examine the MAP set of findings and identify those that could be considered within the ICF framework to illustrate maltreatment and the child welfare system involvement as environmental factors, in conjunction with mental health problems and disabilities as health conditions. While the MAP study did consider selected resilience factors [e.g., self-compassion (50)], and these should be integrated into an ICF framework, they were not the focus of the current exploration.

## Methods

### The Maltreatment and Adolescent Pathways (MAP) Study

A multi-disciplinary research team, guided by a child welfare advisory board, developed and implemented the MAP study. The MAP study collected information about physical health (e.g., sleep quality), mental health (e.g., PTSS), and cognition (e.g., IQ and memory) in adolescents from a large urban CPS system catchment area. The MAP study recruited participants drawn *via* a random numbers table from a CPS agency-provided list of all active caseloads of adolescents (average age = 15.8 years), refreshed every 6 months given the rate of case closures. These included the largest agencies in Canada (50, 51).

Among the 24 MAP sub-studies, 11 were selected for analysis due to their fit with the ICF framework. The fit was determined based on whether the sub-study investigated data on health conditions, contextual factors, and functioning or disability of child welfare-involved youth, so it could be holistically applied to the ICF framework. A description of each included MAP sub-study can be found in **Table 3**. Linking rules were used to connect the concepts in MAP sub-studies with the appropriate ICF categories and to enhance the transparency of the linking process (52, 53). A paediatric consultant, with expertise in the ICF, supported our linking procedures. The MAP sub-studies use a diverse range of measures, as seen in Table 2, and our goal is to link these measure outcomes to ICF categories, with the focus on childhood trauma exposures and adolescent mental health and resilience. The linkage to the ICF was completed by an ICF specialist, child welfare/mental health specialist, and medical student with a background in the study of maltreated youth.

**Table 2:** MAP study measures.

**Outcome**	**Measure(s) used**	**Corresponding code**
Child maltreatment (i.e., physical abuse, sexual abuse, emotional abuse, neglect)	Childhood Trauma Questionnaire [CTQ; (54, 55)] • 70-item self-administered inventory with 5-point Likert-type scales • Addresses five types of maltreatment (emotional abuse, physical abuse, sexual abuse, emotional neglect, and physical neglect) CTQ-short form [CTQ-SF; (56)] • 28-item version of the original CTQ	e310 (immediate family), e398 (support and relationships)
Exposure to intimate partner violence (IPV) (i.e., physical and verbal)	Childhood Experiences of Victimisation Questionnaire [CEVQ; (57)] • Brief 18-item self-report measure of victimisation among adolescents • Addresses peer-on-peer violence, witnessing domestic violence, emotional abuse, physical punishment, physical abuse and sexual abuse	e310 (immediate family), e398 (support and relationships)
Adolescent dating violence (ADV)	Conflict in Adolescent Dating Relationships Inventory [CADRI; (58)] • 46-item self-report questionnaire, with bidirectional questions (victim/perpetrator) • Measures abusive behaviour among adolescent dating partners	d7202 (regulating behaviours within interactions), d7700 (romantic relationships)
Trauma symptoms (i.e., anger, anxiety, depression, dissociation, sexual concerns, PTSS)	Trauma Symptom Checklist for Children [TSCC; (59)] • 54-item self-report of trauma symptoms • Assesses severity of post-traumatic stress and related psychological symptomatology (anxiety, depression, dissociation, anger, sexual concerns)	Health conditions are coded by ICD-10 and not ICF
Psychological distress	Brief Symptom Inventory [BSI; (60)] • 53-item self-report of clinically relevant psychological symptoms in adolescents and adults • Addresses somatization, obsession-compulsion, interpersonal sensitivity, depression, anxiety, hostility, phobic anxiety, paranoid ideation, and psychoticism	Health conditions are coded by ICD-10 and not ICF
Substance abuse (i.e., alcohol misuse, marijuana misuse, use of drugs)	Rutgers Alcohol Problem Index [RAPI; (61)] • 23-item self-administered screening tool for assessing adolescent problem drinking Alcohol Use Disorders Identification Test (AUDIT; (62)) • 10-item questionnaire covering the domains of alcohol consumption, drinking behaviour, and alcohol-related problems CRAFFT (63, 64) • Substance use screening tool for adolescents	d5702 (maintaining one's health)
Sleep disturbances (i.e., taking longer than half an hour to fall asleep, waking up before intended, having non-restorative sleep)	11 self-report questions adapted from standardised sleep disorders questionnaire [SDQ; (65)] SDQ • Short self-rating questionnaire with 18 questions on different sleep problems	b1340 (amount of sleep, b1341 (onset of sleep), b1342 (maintenance of sleep)
Intelligence [i.e., intelligence quotient (IQ)]	Kaufman Brief Intelligence Test [KBIT; (66)] • Individually administered measure of intelligence • Developed specifically for screening and related purposes	Health conditions are coded by ICD-10 and not ICF
Drinking motives	Drinking Motives Questionnaire Revised [DMQ-R; (67)] • Self-reported questionnaire with 20 reasons why people might be motivated to drink alcoholic beverages • Addresses social, coping, enhancement, and conformity motives	b1301 (motivation)
Sexual risk-taking	US Youth Risk Behaviour Survey [YRBS; (68)] • Monitors health-related behaviours among youth and adults (e.g., alcohol use, unhealthy dietary behaviours, inadequate physical activity)	d5702 (maintaining one's health), b1301 (motivation)

Health conditions in the ICF refer to any diseases, disorders, and/or injuries, which may include other circumstances such as stress, ageing, pregnancy, congenital anomality, and genetic predisposition (42, 43). In this study, we identified mental health problems (trauma symptoms and psychological distress) and intellectual disabilities, as measured in the MAP study, such as health conditions. Contextual factors in the ICF include environmental and personal factors, which facilitate or act as barriers to functioning. For the purposes of this study, environmental factors included: child welfare system experience (length of time in the system, foster care or non-foster care, number of residential moves; e5258), caseworker relationship, (number of visits, identification with caseworkers; e360), child maltreatment experience (e310, e398), and exposure to intimate partner violence (IPV; e310, e398) as measured by the Childhood Experiences of Violence Questionnaire [CEVQ; (57)]. Personal factors included sex, age, ethnicity, attachment style, and past experiences (youth-reported history of child maltreatment types) and were coded as personal factors (pf). Activity limitations represent the difficulties an individual would have in executing any given task or action (e.g., walking, eating). Participation restrictions encompass the problems an individual may experience with others during the involvement of life situations (e.g., employment, education). Activity limitations and participation restrictions are considered one component due to their correlated nature. Among youth in child welfare, we have identified substance use (d5702), sexual risk-taking (d5702), and adolescent dating violence (ADV; d7202, d7700) in this component. While ADV may fall under contextual factors (i.e., current victimisation can be environmental; victimization; and/or perpetration history can be personal), they are defined as participation restrictions for the purposes of this study in so far as they reflect the abuse of power and control, with potential limits to the access of other peers and family (socially isolating the partner), and capacity to exit a romantic partnership and/or begin new ones (69). Romantic relationships are an important and under-considered domain of the child welfare system-involved youths' functioning. Especially given the relative lack of close relationships and supportive family for child welfare-involved youth, there may be an early entry into these relationships with peers or older adults (70). Finally, body functions describe the physiological and psychological functions of body systems. We have placed coping motives (b1301) and sleep disturbances (b1340, b1341, b1342) in this component. Coping motives were specifically considered mental functions, as they encompass the motivations to engage in certain behaviours. Body structures (i.e., anatomical body parts) were not explored in this review.

**Figure 1:**
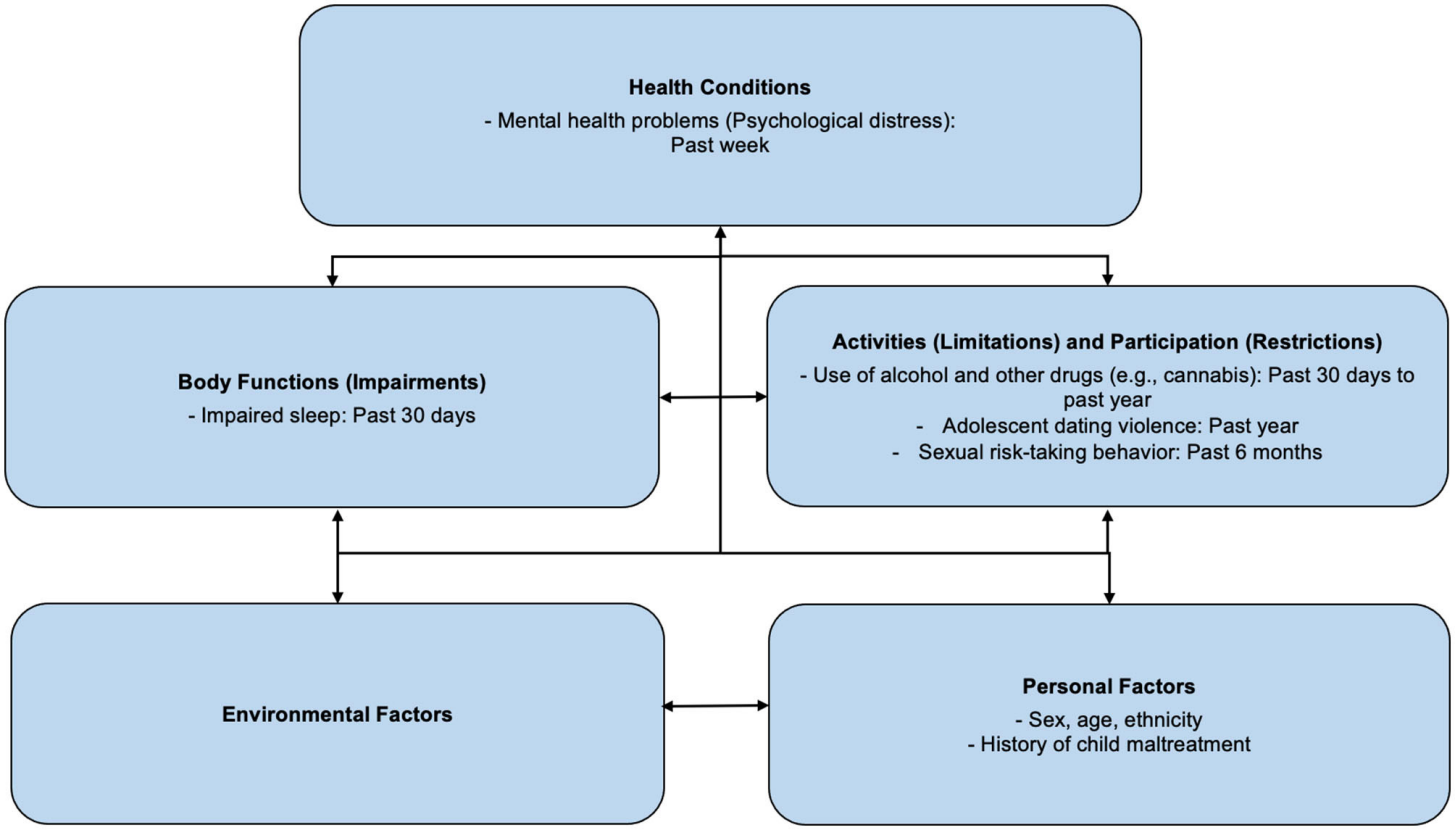
Distal ICF factors: Sexual risk-taking behaviours include early sexual debut, multiple sexual partners historically, a pattern of unprotected intercourse, or sex while under the influence of alcohol or drugs. These may result in negative personal health implications (71). Impaired sleep refers to the presence of difficulty with sleep, which could present itself as sleep onset latency, frequent nocturnal awakenings, or prolonged periods of wakefulness during the sleep period (72).

**Figure 2:**
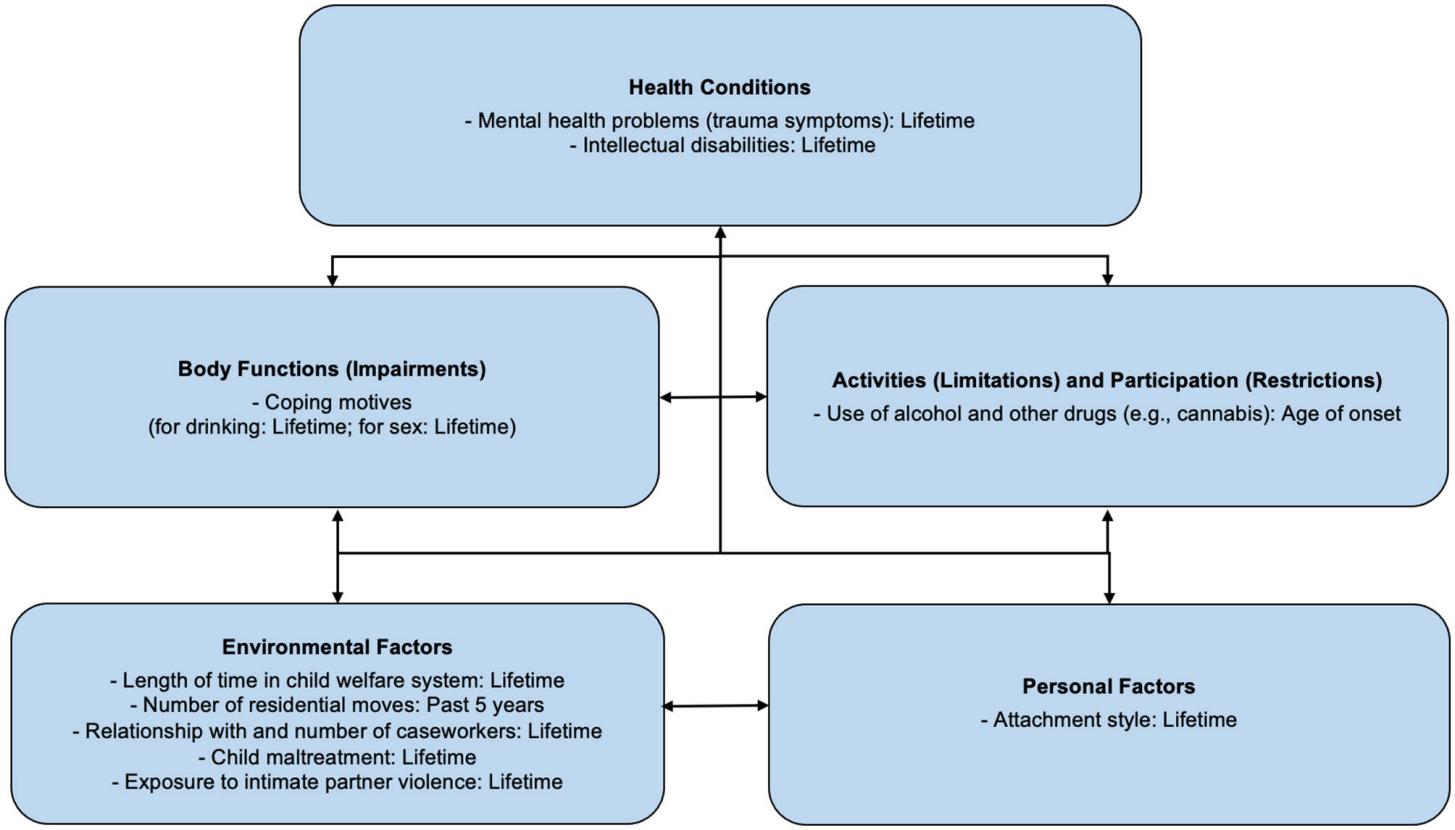
Proximal ICF factors: Coping motives refer to the process governing the choices made towards various coping behaviours, in an effort to manage stress (73). Maladaptive coping motives may lead to health-risk behaviours (e.g., drinking alcohol to cope with negative affect) (74).

The ICF offers a snapshot in time of functioning. Youth in the child welfare system context, however, face instability, such as frequent moves, varying lengths of placements, and recurring victimisation at different time points. Such instability contributes to the need for an analysis of proximal (now or recent) and distal (historical or lifetime) factors that impact the functioning of child welfare-involved youth. For the purposes of this study, proximal factors include factors measured currently or within the past year. Distal factors include factors measured beyond a year or based on frequency, therefore being lifetime (Refer to Figures 1, 2 for our distribution of factors from the MAP study, respectively).

It is important to note that the listed factors measured by the MAP study are not exhaustive of all relevant variables. For example, sexual orientation was reported by youth and fluctuated across MAP assessments over time, which is consistent with the concept of sexual identity exploration, with an overall higher representation of sexual minority status than expected in the population (75, 76). Sex at birth, gender identification, and sexual orientation are currently considered in more expansive categories than were evaluated in the MAP study [e.g., transgender, non-binary, gender queer, etc. (77)]. While sex may be a distal category, gender may be better considered as a proximal variable.

Among the included 11 sub-studies, data were extracted independently by three authors (KK, CM, and JP). For each study eligible for full-text assessment, the number of participants, characteristics of participants (e.g., age, sex, etc.), health conditions, and components of the ICF framework (i.e., environmental factors, personal factors, body functions, activities, and participation) were identified. Any discrepancies regarding these extractions were resolved by discussion among the authors.

## Results

A total of 11 publications were included in our selective review. All 11 publications were published between 2009 and 2019, and used the same MAP study dataset, but varied in their selection of data to analyze (available complete data). Sample sizes ranged from a select sample of 73 (i.e., a sample of youth who completed IQ testing) to its full sample of over 500 subjects. Borderline to mild intellectual disabilities (defined as IQ scores between 60 and 84) were present in 24% of the Weiss et al. (78) study. All analyses reflected more females than males (ranging from 51.8 to 64.4% of participants). Refer to Table 3 for included sub-studies and additional study characteristics descriptions.

**Table 3:** Study characteristics.

**References**	**Aim of study**	**Sample**	**Gender (% female)**	**Mean age (years) [SD]**	**Main findings**
Faulkner et al. (75)	To examine the relationship between experiencing two child maltreatment types (child abuse and neglect [CAN] and exposure to IPV) and two outcomes (substance use and dating violence) in the past year.	*N* = 158 Participants remaining in the MAP study at the two-year follow up and who had initiated dating.	62.7%	17.89 [0.98]	- CAN experiences predicted more frequent dating violence perpetration and greater alcohol problems, indirectly via anger. - Exposure to caregiver IPV was associated with dating violence, indirectly via anxiety and anger. - Exposure to caregiver IPV was associated with marijuana use, indirectly via anxiety and dissociation. - Exposure to caregiver IPV was associated with past year occurrence of both marijuana use and dating violence, indirectly via anger
Goldstein et al. (79)	To develop a framework that identifies risk of alcohol problems and the likelihood of benefiting from preventive interventions among child welfare-involved adolescents.	*N* = 202 Participants who had consumed alcohol in the past year and had complete data on the variables of interest.	54.5%	15.93 [1.03]	- Child maltreatment was significantly and positively associated with alcohol problems and with coping motives. - Coping motives were significantly and positively associated with anxiety symptoms and with alcohol problems. - Increased anxiety symptoms were associated with more alcohol problems for adolescents with high coping motives compared to low. - Increased depression symptoms were associated with fewer alcohol problems among those with high coping motives compared to low.
Goldstein et al. (80)	To examine the contribution of post-traumatic stress symptoms to substance use and substance related problems among child welfare-involved adolescents and emerging adults.	*N* = 253 Participants who had complete data for variables of interest at the initial, 6-month and 1-year assessment points.	61.4%	16.87 [1.04]	- All maltreatment types were positively and significantly associated with all trauma symptoms in TSCC measure (except for the association between sexual abuse and anger). - Child maltreatment and dissociation were positively associated with using a greater number of illicit drugs (other than marijuana) in the past year. - Anger and dissociation were positively associated with alcohol use and drug problems. - Sexual concerns were negatively associated with drug problems.
Hudson et al. (81)	To examine the gender differences within the links between CSA and alcohol problems in adolescence, via potential emotion-focused mechanisms, among child welfare-involved youth.	*N* = 301 Participants who had complete data on CSA, negative emotion symptoms, and problem drinking questionnaire items.	56%	16.4 [1.0]	- CSA was positively associated with increased levels of anxiety, depression, and anger. - CSA was indirectly associated with problem drinking, via anxiety and anger among female adolescents (full mediation by negative emotions). - CSA was indirectly associated with problem drinking, via anger (partial mediation by negative emotions).
McPhie et al. (51)	To examine the relationship between child maltreatment history and sleep quality among adolescents.	*N* = 73 Participants in the initial and 2 year time points with complete data on variables of interest.	64.4%	15.9 [1.06]	- Initial severity of child maltreatment predicted sleep problems 2 years later. - Psychological distress fully and positively mediated the relationship between child maltreatment and sleep problems.
Park et al. (82)	To examine how PTSS and coping motives mediate the association between child maltreatment and alcohol use.	*N* = 564 Participants with complete data on childhood trauma, PTSS, drinking motives, and alcohol misuse at initial data collection and 6-month follow-up.	53.7%	15.9 [1.1]	- Child maltreatment was positively correlated with PTSS. - PTSS were positively correlated with coping motives. - Coping motives were positively correlated with alcohol misuse. - PTSS and coping motives mediated the relationship between child maltreatment and alcohol misuse via a serial mediation (i.e., not as single mediators).
Tanaka & Wekerle (83)	To examine self-reports of ADV victimisation and perpetration among child welfare-involved youth.	*N* = 341; 110 Participants who completed the ADV measurement at initial data collection; participants who completed the ADV measurement across all data points.	54%	15.8 [1.1]	- ADV verbal/emotional abuse was most highly endorsed. - ADV sexual abuse was least endorsed. - No significant difference in ADV perpetration and victimisation scores and prevalence across genders. - Male ADV victimisation was higher for non-foster care youth compared with foster care youth; male ADV perpetration did not differ. - Females did not differ based on CPS status. - 33.6% of youth have never been exposed to ADV from initial data collection to the 2 year follow up. - 46.4% reported ADV 2+ assessment points, up to the 2 year follow up.
Waechter et al. (84)	To examine the relationship between cannabis use and self-reported identification with a caseworker among Indigenous and non-Indigenous adolescents.	*N* = 476 Participants who self-identified as Indigenous or other.	53%	15.8 [0.99]	- Indigenous youth did not differ from the non-Indigenous youth on their child maltreatment types, IPV, PTSS, cannabis usage, nor identification with CPS workers. - Indigenous youth who reported low identification with their caseworker were 5.47 times more likely to have ever used cannabis in the past 12 months compared to non-Indigenous youth with low identification.
Weiss et al. (78)	To examine attachment styles and ADV in child welfare-involved adolescents with borderline-to-mild intellectual disability.	*N* = 167 Participants who completed intelligence testing and experienced clinically significant maltreatment histories.	58%	15.8 [0.98]	- Adolescents with borderline-to-mild intellectual disabilities reported significantly more ADV victimisation and perpetration than adolescents with average IQ. - Rates and severity of child maltreatment histories were similar across adolescents with intellectual disabilities and with average IQ. - Avoidant attachment style significantly predicted ADV victimisation and perpetration, particularly among adolescents with lower IQ.
Wekerle et al. (76)	To examine sexual motives, CSA, and risky sexual behaviour among child welfare-involved youth. To also evaluate motivations for sexual behaviour as a potential mechanism from CSA to risky sexual behaviour among adolescents.	*N* = 297 Participants who endorsed being sexually active (defined as having had sexual intercourse at the time of initial assessment).	57.6%	15.83 [1.04]	- CSA was associated with severity of all child maltreatment types for both genders. - CSA was associated with witnessing emotional IPV for females only. - CSA was associated with more sexual risk taking, particularly among males, compared to youth with no CSA experiences. - CSA was associated with greater coping motives, which in turn was associated with increased sexual risk-taking.
Wekerle et al. (85)	To consider the predictive value of childhood emotional abuse to understand PTSS and ADV. To also assess PTSS as a mediator between childhood emotional abuse and ADV.	*N* = 402 Participants who had data on childhood maltreatment histories, PTSS and ADV at initial data collection of the MAP study.	51.8%	16.3 [0.99]	- Emotional abuse significantly predicted both PTSS and dating violence among males and females. - PTSS significantly mediated the relationship between male emotional abuse and ADV perpetration. - PTSS significantly mediated the relationship between female emotional abuse or physical abuse and ADV victimisation.

### Contextual Factors

The ICF model postulates a dynamic interaction across health conditions, contextual factors, and functioning. The influence of personal and environmental factors presents across the different domains of functioning and disability. Our focus is on child maltreatment as an environmental factor.

#### Maltreatment Experiences

Maltreatment and Adolescent Pathways (MAP) youth reported high rates of maltreatment distally. In McPhie et al. (51), 78.1% of the participants reported one or more types of maltreatment histories above the Childhood Trauma Questionnaire [CTQ; (56)] clinical cut-offs for severity. Rates and severity of child maltreatment histories did not differ based on participant IQ, or Indigenous identity (78, 84). In Hudson et al. (81), 37% of participants self-reported CSA histories. CSA histories emerged as a particularly impactful form of maltreatment: it was related to the severity of all types of child maltreatment for both sexes (76). Faulkner et al. (75) investigated both maltreatment histories and lifetime exposure to IPV. In this analysis, 64.6% of participants reported two or more types of childhood maltreatment histories. Respectively, 60.8 and 38.6% of individuals witnessed lifetime verbal violence and physical IPV, according to the CEVQ. High levels of poly-victimisation rates of maltreatment were confirmed in this child welfare-involved sample.

#### Child Welfare System Experiences

Most MAP youths had received long-term services, in the 5–6-year range, with mid-childhood (ages 9–12), as the most common entry time into care. Multiple placements were the norm: youth in the MAP study moved in the range of 0–5 times in the past 5 years (averaging at 2.1 times). The provision or level of treatment or referral was not known; in this Ontario sample, there were mandated caseworker visits every 90 days to the youth. However, caseworker compliance and the length and nature of caseworker visits were not known. Only identification with caseworkers was investigated in Waechter et al. (84), and it did not differ between Indigenous and non-Indigenous child welfare-involved youth. In this study, the question of whether positive identification (as compared to negative identification) with a caseworker was considered to understand to some degree the nature of the relationship.

### Health Conditions

#### Mental Health Problems

In terms of the relationship of maltreatment types to health conditions, there was a range of significant findings across mental health conditions, with child emotional and sexual abuse showing higher levels of adolescent concern. McPhie et al. (51) found that 28.2% of the sample reported one or more symptoms of psychological distress within the clinical range in the past week on the BSI measure, which is a proximal distress measure [domains of somatization, anxiety, depression (60)]. Goldstein et al. (80) and Park et al. (82) reported that all types of child maltreatment histories (i.e., physical abuse, sexual abuse, emotional abuse, physical neglect, emotional neglect) had positive, significant correlations with the trauma symptoms, in terms of the TSCC (59) total score, as well as the subscales of PTSS, dissociation, and sexual concerns. As the TSCC does not specify a timeframe, it may be conservatively regarded as reflecting lifetime, rather than current trauma symptoms. Emotional abuse history was a strong predictor of TSCC total score in Wekerle et al. (85), when all other forms of historical maltreatment were taken into account statistically, indicating a unique contribution. CSA history was associated with increased anxiety, depression, anger, and PTSS (81). Trauma symptoms did not differ based on Indigenous identity (84).

### Activity Limitations and Participation Restrictions

#### Adolescent Dating Violence (ADV)

Adolescent dating violence (ADV) was assessed in the timeframe of a romantic partnership in the past year, and a dating relationship was defined as extending 2 or more weeks, based on relationship prevalence information (58). Over 60% of the MAP sample (aged 14–17 years old) endorsed having begun or been in a dating relationship. While the majority of child welfare-involved youth reported being in a relationship, many were also not reporting a relationship status. Of a 158-participant sample, 77.2% reported perpetrating verbal/emotional ADV, 36.7% threatening violence, 32.9% using physical violence, and 11.4% using sexual violence (75). In Wekerle et al. (85), among MAP dating youths, the majority experienced either form of ADV (i.e., over 60% of females; over 40% of males), noting that perpetration and victimisation behaviours total scores were positively correlated. Male participants with non-foster care status had higher ADV victimisation, as compared to foster care male youth, whereas ADV perpetration did not differ between sexes or foster care status (83). ADV over the MAP assessments were considered in Tanaka et al. (83). From a longitudinal perspective, the minority of child welfare-involved youth (33.6%) were never exposed to ADV, from initial data collection to 2-year follow-up. About 46% of youth reported experiencing repeated ADV (2+ times across assessments), although whether the romantic partner remained the same or varied could not be determined. In terms of types of ADV, verbal and/or emotional abuse was most highly endorsed, and sexual abuse was least endorsed. This is consistent with the view of ADV as a potential participation restriction.

Child maltreatment histories, trauma symptoms (i.e., anger and anxiety), intellectual disabilities, and foster care status were associated with both victimisation and perpetration of ADV. In Wekerle et al. (85), considering sex differences, trauma symptoms mediated the relationship between emotional abuse and ADV perpetration for males, as well as the relationship between emotional abuse or physical abuse and ADV victimisation for females. ADV perpetration was predicted by child maltreatment histories, with adolescent anger functioning as a mediator. In this context, trauma-related anger is, in part, explanatory for aggression against a partner; it should be noted, though, that some of this behaviour may reflect a violent dynamic of back-and-forth, potentially escalating aggression, as the CADRI does not capture the interplay but only presence or frequency. Considering ADV perpetration, exposure to caregiver IPV was a predictor, indirectly, *via* trauma-related anxiety and anger (75).

Intellectual disabilities and adolescent attachment styles also played a role in ADV. Attachment style is conceptualised as a generally consistent variable over time and was measured by Attachment Security Ratings (86). Avoidant attachment denotes discomfort towards long-term relationships (87), and an avoidant style reflects a tendency to avoid emotional closeness. Thus, both intellectual disability and attachment style would be historical, although the attachment style in adolescence is not crystallised and would be expected to change, for example, with intervention (86). Adolescents with borderline to mild intellectual disabilities reported significantly more experiences of ADV victimisation and perpetration, compared to adolescents with average IQ. Particularly among adolescents with lower IQ, having an avoidant attachment style significantly predicted both ADV victimisation and perpetration, indicating the risk in avoidance as a style for interacting with others (78). The secure attachment style reflects flexible responding, and confidence in the other interactant to provide a secure base for development, support, and exploration. An avoidant interpersonal style may be protective in terms of exposure to dangerous persons and situations; however, it represents a limitation in experiencing emotional connectedness.

#### Substance Use

Histories of child maltreatment, trauma symptoms (i.e., anger, anxiety, dissociation, and sexual concerns), and Indigenous identity had significant impacts on substance use among MAP study participants. Greater alcohol problems were predicted by child maltreatment histories and were mediated by adolescent anger (75). In the same sample, 38.6% of youth reported at least one incident of binge drinking in the last 30 days, 40.3% of youth reported alcohol use in the past year, and 10.1% of youth met the cut-off for the development of a drinking problem. Given that the legal drinking age in Ontario (age 19) was outside of all MAP study participants, these numbers are concerning. In Hudson et al. (81), CSA history was associated with problem drinking and was partially mediated by anger for male adolescents, and fully mediated by anxiety and anger for female adolescents.

A significant number of MAP youth reported drug use. Faulkner et al. (75) found that 45.6% of the sample reported cannabis use in the last 30 days. In addition, 19.3% of youth in child welfare reported using illicit drugs. Similarly, child maltreatment history was significantly and positively associated with alcohol problems (79). In Goldstein et al. (80), anger and dissociation symptoms were positively associated with alcohol and drug problems (80). Child maltreatment histories and dissociation were both positively associated with using a greater number of illicit drugs in the past year (80). With regards to cannabis usage specifically, associations were found with exposure to caregiver IPV, mediated by anxiety, dissociation, and anger (75).

Cannabis usage was not dependent on Indigenous identity, as Indigenous and non-Indigenous youth reported no differences in Waechter et al. (84). However, Indigenous youth who reported low positive identity with their caseworker were 5.47 times more likely to have used cannabis in the past 12 months, compared to non-Indigenous youth with a low caseworker identification (84).

#### Sexual Risk-Taking

Sexual health and sexual risk-taking are interconnected. Early entry into sexual relationships was notable for MAP males (25% before age 13), and sexual health practises varied (e.g., 12% of MAP females reported that they never used protection in intercourse) (85). Sexual risk-taking indicators formed a risk-taking score and were assessed as age at first sexual intercourse, alcohol/drug use before engaging in sex, condom use, positive testing for sexually transmitted infections, and a total number of sexual partners. Wekerle et al. (76) reported a significant overall relationship between CSA history and adolescent sexual risk-taking; for instance, adolescents with a history of CSA were more likely than those without a history to have had sex with multiple partners.

### Body Function Impairments

#### Coping Motives

Coping motives predominantly acted as mediators for the relationships across child maltreatment histories, trauma symptoms, and alcohol misuse or sexual risk-taking. For example, coping motives (e.g., to become more sociable, comply with peer pressure, forget about worries, feel good) for drinking were significantly and positively associated with child maltreatment and anxiety (79). For those with high maladaptive coping motives, greater anxiety was associated with more alcohol problems, whereas lower depression was associated with fewer alcohol problems. In Park et al. (82), both trauma symptoms and coping motives mediated the positive relationship between child maltreatment and alcohol misuse *via* serial mediation (82), showing that both are important for understanding adolescent substance use.

Child sexual abuse (CSA) was also associated with a greater number of maladaptive coping motives, as compared to non-CSA MAP study youth. CSA youth were significantly higher on having sex to cope with negative emotions, in both males and females than non-CSA youth. CSA history was also associated with greater maladaptive coping motives for sex (i.e., having sex in order to gain peer and/or partner approval), which in turn were associated with increased sexual risk-taking (i.e., a higher number of partners, not using protection during sex, using alcohol or drugs before sex) (76).

#### Sleep Disturbances

Sleep disturbances in the past 30 days were analysed by one study and measured with 11 self-report questions adapted from standardised questionnaires. McPhie et al. (51) found that the most reported sleep problems include taking longer than 30 min to fall asleep (61.6%), waking up before intended (46.4%), and having non-restorative sleep (38.4%). It was also reported that the severity of childhood maltreatment was predictive of sleep disturbances, where psychological distress functioned as a mediator. As the distress measure tapped proximal distress, it suggests that such issues as trauma symptoms, anxiety, and anger, as previously noted, are current candidates in determining body or physical regulation.

## Discussion

Multiple outcome research from a large child welfare system dataset with multiple types of mental health and functioning measures support the utility of an ICF approach to understanding the holistic health of maltreated youth who come to the attention of formal child welfare systems. An ICF approach provides added value in directing assessment attention (e.g., adolescent dating violence, sexual risk-taking), in an ongoing fashion, and incorporates domains of clinical relevance (e.g., coping motives), not typically included in assessment models. As indicated in this study, unique and significant challenges are faced by youth in child welfare settings. A distal vs. proximal model seems important to understand the interplay of factors and the changing landscapes of maltreated adolescents receiving child welfare services. Histories of child maltreatment were consistently and positively correlated with mental and physical health conditions (i.e., trauma symptoms, intellectual disabilities) and functioning problems (i.e., substance use, ADV, sleep problems, sexual risk-taking). For example, all maltreatment types and trauma symptoms presented positive relationships, showing the important linkages and mediator role of trauma symptomatology and, hence, the need to assess adolescent trauma symptomatology. In terms of specific types, exposure, and certain maltreatment forms (IPV, CSA, and emotional abuse) emerged as impacting functioning more significantly. Histories of emotional abuse were predictive of both PTSS and ADV in the past year for male and female youth (85), yet, relatively little attention is given to it clinically (88). The practitioner under-attention to emotional or psychological maltreatment prompted a statement from the American Paediatrics Association to review findings and identify that it needs to be considered alongside and independently from the more often considered physical and sexual abuse, in terms of assessment, prevention, and the potential utility of trauma-focused treatments (89).

While the popular CTQ measure was primarily used for investigating historical child maltreatment in the MAP sub-studies, the CEVQ filled the gap of providing information on exposure to IPV. The historical childhood exposure to IPV as a distal factor was significantly linked to the proximal experience of ADV, in which trauma symptomatology emerges as a bridging factor. ADV emerged differently over time, such that at a mid-adolescent timepoint (i.e., the MAP initial age described youth, on average, ranging from 15 to 16 years), the minority of youth reported ADV. However, with a view from the initial assessment to the 2-year follow-up, where youth are in late adolescence (ages 17–18 years), most youth had experienced some form of ADV. High rates of ADV perpetration and victimisation indicate the need for interventions on relationship skill building and safety skills. Foster care and non-foster care youth vary in their access to resources, so making sure these interventions are equitably distributed is essential. This is especially important when considering the increased prevalence of ADV victimisation among non-foster care male youth, given that greater service provision typically is directed to foster care youth (83). Thus, when it comes to relationship violence, a distal by proximal view seems important and needs to be part of the ongoing inquiry during caseworker visits and in adolescent medicine.

The ICF framework accommodates well the interplay between environmental factors with health conditions. Given the placement disruptions of youth in the MAP study, the risk for disrupted attachments, trauma, and instability in social networks seem more likely. Analyses of child welfare statistics in Canada show that youth in care face increased environmental turbulence, such as multiple placement moves (90). A greater number of moves has been associated with difficulties in building consistent relationships in new schools and community neighbourhood settings, as well as the development of more severe trauma symptoms (91, 92). Practitioners are encouraged to continue checking such quality-of-life factors, particularly for those involved in the child welfare system. Given the consistent mediator role of trauma-related emotionality (distress, anxiety, anger) for both males and females, supporting youth to be more fully engaged in the decision-making is not only a rights-based expectation (i.e., sustainable development goal 16, right to be free from all forms of violence) but also a principle of trauma-informed care (93). During adolescence, it is critical for youth to grow and develop in supportive environments, with ample opportunities to connect safely, access resources, and practise adaptive emotion regulation skills. Youth with histories of greater poly-victimisation are more likely to experience maltreatment while in foster care, compared to those with fewer maltreatment experiences (94), and care needs to be prioritised to prevent such re-victimisation and traumatization. The medical home may be an important point of continuity of care.

It was found that child welfare-involved youth with borderline-to-mild intellectual disabilities reported significantly greater dating violence victimisation and perpetration, compared to youth with average IQs (78). Children with disabilities are at greater risk of experiencing child maltreatment, with three times the higher prevalence of maltreatment than the typically developing population (95). When considering the whole child welfare system, ~50% of children investigated by CPS had a developmental disability(ies) (95). More concerning is the understanding that maltreatment rates are likely higher than reported, as those with disabilities may experience communication difficulties that hinder disclosure or self-report to caseworkers or practitioners.

Weiss et al. (78) further detected the role of attachment style, where avoidant attachment styles correlated with both ADV victimisation and perpetration, particularly among adolescents with low IQ. Children with maltreatment histories are less likely to seek abusive attachment figures (e.g., parents, caregivers) for support or comfort, which can, in turn, lead to increased fears related to attachment figures and, hence, the development of avoidant attachment styles (96). Insecure attachment models, which begin at an early age, likely carry out into adolescence in terms of developing a stylistic way of relating which, in turn, can impact the ability to engage in healthy relationships (97). It may, therefore, be useful to consider the maltreated, child welfare-involved youth's experiences with close relationships, and how that influences their approach/avoidance styles. Youth with an avoidant style may not make the connexion to how this style of interaction expresses in a romantic relationship, for example, and understanding this dynamic may help to direct the clinician's anticipatory guidance approach. Research has shown the potential of modifying attachment styles *via* treatment and can therefore be a goal of intervention for child welfare-involved youth, who are at greater risk for ADV involvement (98).

Child maltreatment experiences, trauma symptoms, and coping motives were frequently linked together in the MAP sub-studies. Emotion regulation emerges as a core issue in understanding both the tendency towards emotion avoidance (e.g., avoidant attachment style) and the mediation by trauma-related symptomatology. The MAP sub-study findings suggest a coherence in the core issue of emotion regulation and coping across diverse outcomes (i.e., ADV, substance use, or sexual risk-taking). Substance use across child welfare settings stood out as a particular concern. Similar to childhood IPV exposure and later dating violence, the social learning theory argues how youth can learn positive or negative behaviours from observing others that inform the experiences of victimisation and later coping strategies (99). If substance use or IPV is prevalent in initial households, as these tend to overlap [e.g., (100)], this could be a contributing factor to later substance use and ADV problems. Indeed, MAP youth with CSA histories identified motivations to have sex as focused on a way to cope with negative affect. Alternatives to coping with trauma-related negative affect and addressing emotion regulation challenges (e.g., emotional reactivity to threat/perceived threat, delayed emotional responding with dissociation) may be an important clinical goal. These higher rates of substance use among MAP youths are consistent with previous studies. For example, youth with four or more adverse childhood experiences are approximately two, seven, five, and 11 times more likely to engage in smoking, alcoholism, illicit drug use, and injected drug use, respectively, than those with fewer ACEs (101). Substance use was also often associated with coping motives, which we considered an impaired body function. Drinking, for example, may serve as a tension-reduction mechanism to cope with feelings of anxiety and, in turn, better allow youth to participate in social events in dampening social anxiety. Such coping may work in tandem with the impact of child maltreatment histories, which are also associated with the inability to develop healthy coping motives. These coping motives were also present in reasons for having sex, particularly among those with CSA histories. Adaptive coping and social skill development are important targets to consider for child welfare system-involved youths.

### Clinical Implications

For the practitioner, the ICF model is valuable in guiding service provision to foster care and/or maltreated youth. Currently, the HEADSS (Home; Education and employment, eating and exercise; Activities and peer relationships, social media; Drug use, including prescribed medications, cigarettes, vaping, alcohol and other drugs; Sexuality and gender; Suicide, self-harm, safety, and spirituality) assessment is used as a psychosocial assessment for adolescents by health practitioners (102). In reference to child welfare-involved youth, however, it is a missed opportunity to understand the why behind health-risk behaviours, such as maladaptive coping. For example, coping motives were significant in correlating with alcohol problems and sexual risk-taking behaviour (79, 82). Both behaviours are being engaged in for managing negative emotions, which highlights the need for services that directly target the management of emotions (i.e., emotion literacy, adaptive emotion expression, managing distress, alternatives to acting-out behaviours, and resilience strategies). Interventions that support new routines in emotion regulation would seem important, such as Trauma-focused Cognitive Behaviour Therapy, which addresses PTSS with cognitive-behavioural principles (103); and interventions based on behavioural perspectives, which focuses on understanding antecedents and consequences of actions (104). More research is needed to determine which interventions are most effective for child welfare-involved youth particularly. The interrelated nature of the ICF model further reinforces the ways in which contextual factors and health conditions interact to influence functioning, appropriate to the complex nature of youth experiences within child welfare.

Additionally, it may be practical to consider the “medical home” when providing care for child welfare-involved youth (105). A medical home is an approach that integrates patients, families, clinicians, and medical staff, to provide comprehensive primary care. They are well-known in their ability to conduct ongoing screening and facilitate longitudinal relationships, which are critical for the turbulent environment of child welfare-involved youth. The medical home can stand as a source of stability. Research has shown that youth with special healthcare needs (i.e., requiring additional health-related services due to increased risk of developmental, physical, emotional, and/or behavioural conditions) benefit from the medical home, as seen by improvements in health, school, and work attendance, as well as access to care (106). The medical home, as an environmental factor may, therefore, facilitate functioning in this population. In a meta-analysis, it has also been found that communication with parents about safe sex can act as a protective role in adolescent safer sex behaviour (107). Professionals in the medical home, or caregivers in the child welfare system, can have these conversations or support guardians in this regard, as prevention against sexual risk-taking and compromised sexual health.

It is a given that clinicians interacting with youth from the child welfare system be well-versed in current literature in the child maltreatment area. For developing clinicians and trainees, various resources and guidelines currently exist for clinicians to use in the care for child welfare-involved youth, including The Encyclopaedia of Early Childhood Development (108) and the VEGA (Violence, Evidence, Guidance, Action) Project (109), as open access options for information and training about detecting and responding appropriately to child maltreatment and family violence. Similarly, there are several guidelines and tools for mental health treatment, such as the WHO Mental Health Gap Action Program (110) and the American Academy of Paediatrics primary care tools (111). Using such tools can support better care for vulnerable youth.

### Strengths and Limitations

Strengths of this review include the use of the ICF to analyze the health and functioning of child welfare-involved youth. As seen in the MAP sub-studies, multiple factors were connected and significantly related to one another. Trauma symptoms often functioned as mediators (e.g., relationships between sleep problems and child maltreatment histories; exposure to IPV and substance use, etc.), which aligns with the multi-faceted nature of the ICF model.

Limitations include the incompatibility between the measure timelines and the ICF model. The ICF is a snapshot, meaning it represents the functioning of an individual at a specific time; comparatively, the MAP study measures included a wide range of timeframes. For example, child maltreatment was measured by the CTQ-SF, which reports maltreatment history “while growing up” without a specific time frame; in contrast, the CEVQ reports the frequency of IPV and maltreatment experiences more specifically (e.g., between grades 1–5, grades 6–8, happening now, etc.) providing better quantification of distal vs. proximal influences. Moving forward, questionnaires should be standardised in a way that can disentangle proximal vs. distal time points, and graphical databases may be implemented to visualise the various timelines.

The MAP sub-studies were also ambiguous in using the terms, “gender” and “sex,” interchangeably. This may be attributed to the publication year of some studies, during a time in which definitions of gender and sex definitions were not properly delineated. While sex was analysed in the MAP sub-studies, it can be insightful to expand research to include a range of genders, as gender and sexual minorities may be at greater risk of adverse outcomes in child welfare settings (49). Similarly, future steps should involve further analysis of the disproportionate representation of Indigenous youth in child welfare (112). Family and community may both be significant factors of resilience in Indigenous youth, who strongly value and uphold relationships, which can better contribute to appropriate activities and participation in daily life. The MAP study was limited in its analysis of this population, but even then, was able to detect the importance of relationships, as Indigenous youth who experienced good caseworker relationships were significantly less likely to engage in cannabis use. In Ontario, if a youth self-identifies as Indigenous, they are directed to the Indigenous child welfare agency, if available in that locale. The over-representation of diversity sub-groups, and its potential overlap with socioeconomic disadvantage, is an important ongoing area that child welfare continues to contend with, and is addressed with different service models.

## Conclusions

The environment in which a youth is embedded is critical to their ability to function and navigate life in a healthy manner. This is even more true for youth in child welfare settings, experiencing a high rate of adversity and ongoing risk. Any interventions, or solutions, to promote the well-being of child welfare-involved youth ought to address the various interrelated factors involved, which the ICF framework is well-suited to achieve.

## Author Contributions

KK, CM, JP, and CW contributed to conceptualising the review topic and aim. KK, CM, and JP extracted and analysed data. KK wrote the majority of the first manuscript draft. All authors contributed to writing parts of the manuscript, provided feedback on an ongoing basis, revised the manuscript, and approved the submitted version.

## Funding

This review was funded, in part, by the Canadian Institutes of Health Research Team (grant no. TE3-138302) and on a CIHR Indigenous Gender and Wellness (grant no. 171382) (both to CW as principal investigator).

## Conflict of Interest

The authors declare that the research was conducted in the absence of any commercial or financial relationships that could be construed as a potential conflict of interest.

## Publisher's Note

All claims expressed in this article are solely those of the authors and do not necessarily represent those of their affiliated organizations, or those of the publisher, the editors and the reviewers. Any product that may be evaluated in this article, or claim that may be made by its manufacturer, is not guaranteed or endorsed by the publisher.
